# An insight to better understanding cross border malaria in Saudi Arabia

**DOI:** 10.1186/s12936-023-04467-9

**Published:** 2023-02-02

**Authors:** Shaymaa A. Abdalal, Joshua Yukich, Katherine Andrinoplous, Steve Harakeh, Sarah A. Altwaim, Hattan Gattan, Brendan Carter, Mohammed Shammaky, Hatoon A. Niyazi, Mohammed H. Alruhaili, Joseph Keating

**Affiliations:** 1 Department of Medical Microbiology and Parasitology, Faculty of Medicine, King Abdulaziz University and King Abdulaziz University Hospital, Jeddah, Saudi Arabia; 2 Tulane University School of Public Health and Tropical Medicine, New Orleans, LA USA; 3 Saudi Arabia Ministry of Health, Jazan, Saudi Arabia; 4 Department of Medical Laboratory Sciences, Faculty of Applied Medical Sciences, King Abdulaziz University, Jeddah, Saudi Arabia

**Keywords:** Malaria, Border malaria, Saudi Arabia, Human movements

## Abstract

**Background:**

Border malaria is a major obstacle for the malaria elimination in Saudi Arabia. Today, the southern border of Saudi Arabia is a region where malaria cases are resurging, and malaria control is dwindling mainly due to the humanitarian crisis and the conflict in Yemen. This study analyses the current border malaria epidemiology along the southern border of Saudi Arabia from 2015 to 2018.

**Methods:**

All reported cases maintained by the malaria elimination centres in Aledabi and Baish, Jazan Province, Saudi Arabia, from 2015 to 2018 were analysed to examine the epidemiological changes over time. Pearson’s Chi-Square test of differences was utilized to assess differences between the characteristics of imported and local causes and between border cases. A logistic regression model was used to predict imported status was related to living along side of the border area.

**Results:**

A total of 3210 malaria cases were reported in Baish and Aledabi malaria centres between 2015 and 2018, of which 170 were classified as local cases and 3040 were classified as imported cases. Reported malaria cases were mainly among males, within the imported cases 61.5% (1868/3039) were residents of the border areas.

**Conclusions:**

Given the complexity of cross-border malaria, creating a malaria buffer zone that covers a certain margin from both sides of the border would allow for a joint force, cross-border malaria elimination programme. To initiate a malaria elimination activity and cases reported as belonging to this zone, rather than being pushed from one country to the other, would allow malaria elimination staff to work collaboratively with local borderland residents and other stakeholders to come up with innovative solutions to combat malaria and reach malaria-free borders.

## Background

Saudi Arabia had come a long way since 2004 when the National Malaria Programme emphasized that “Saudi Arabia re-expressed its elimination ambitions “of malaria [1]. The sustained control measures consisted of (a) targeting high-risk areas with sustained preventative measures, such as long-lasting insecticidal nets (LLINs) and indoor residual spraying (IRS); (b) timely diagnosis and treatment for all malaria infection cases; (c) reactive surveillance with appropriate treatment and integrated vector control; (d) active case detection at borders with screening and providing preventive and curative malaria treatment [1] resulted in eliminating malaria from many parts of the country, except the southern border region.

Despite the high Kingdom’s commitment to achieving elimination, containment and controlling malaria transmission along its southern border presents a major challenge to reaching the elimination goal [2]. As the incidence of locally acquired malaria in the southern region (Jazan and Aseer regions) steeply declined from (123.8 per 100,000 population to 41.0 per 100,000 population) in 2000 to (below 2.5 per 100,000 population) in 2015. However, a recent dramatic rise in local case incidence rate was observed in 2016 and 2017 across the southern border to 7.5 per 100,000 population and 4.8 per 100,000 population, respectively, and such a jump in the incidence rate was deeply concerning [3]. Likewise, the proportion of cases classified as imported increased to approximately more than 95% of the reported malaria cases [4]. Recently, more than a third of all imported infections detected in the country can be traced back to Yemen [1, 3]. Although Yemen has always been regarded as a source of malaria importation, the war-torn state of the country has caused further worsening of the humanitarian crisis, and, coupled with the rapid economic and agricultural development in Jazan, led to huge population movements to secure jobs or escape the ongoing war [5].

Malaria testing and treatment are available free of charge to everyone regardless of their status through the national health system and through the private sector. The first-line treatment of uncomplicated *Plasmodium falciparum* malaria is 3 days of artesunate (AS) + sulfadoxine–pyrimethamine (SP) and a single low dose of primaquine, according to national malaria policy [6].

Makkah in Saudi Arabia constitutes a major risk for imported malaria. Such a risk is mainly due to the influx of pilgrims and others to perform Hajj and Umrah every year. Many of those from endemic areas with malaria. Additionally, many Saudis visit places that are endemic for malaria for recreational and/or business activities every year. The results of a retrospective study conducted between 2014 and 2015 indicated that 235 malaria cases were reported. Among the cases reported, only 3 cases were Saudi, and the other 232 were not. The infected Saudis traveled to endemic areas. The majority of these cases were (79.6%) caused by *P. falciparum*, and the remaining was caused by *P. vivax*. Most of the cases were from Pakistan, Chad, Sudan and Nigeria. Sorting of mosquito populations revealed the absence of malaria vectors in Makkah District [7].

Based on the 2020 WHO malaria report, it has been estimated that there are approximately 18.8 million cases of malaria reported in Yemen due to their living in areas at risk where the prevalence of malaria transmission is high, resulting in one million cases per year [7]. These are some of the epidemiological evidence of the malaria burden in the study sites and the bordering areas of neighbouring countries.

Mathematical modelling shows how human movement between low malaria-endemic areas and high malaria-endemic areas resulted in regular importation of malaria parasites into areas that would otherwise be non-endemic and can become an epicentre for ongoing transmission [8–12]. Furthermore, studies from many countries aiming to eliminate malaria and are bordering higher burden neighbouring countries indicated that transmission persisted as a consequence of a high number of imported cases [13–17]. Churcher et al. [18] showed evidence that controlled non-endemic malaria areas (which indicates that malaria transmission with current control intervention cannot be self-sustained locally if importation ceased) has been reached once 32% to 48% of reported malaria cases are imported cases.

Given that controlling malaria transmission in the southern border is a complex, multifaceted issue, there is an urgent need to understand malaria trends, identify the subgroups population that are at the highest risk of malaria infection, and what risk factors influence malaria infection the most. While studies on the changes in epidemiological malaria characteristics during the stages of disease control and elimination stages have been conducted in Saudi Arabia, few of these studies investigated the changes in malaria epidemic characteristics from the control stage (2005–2010) [2, 4, 19–24] to the elimination stage (2011–2016) [21] in Jazan Provinces.

This study was undertaken to:

- Describe the epidemiological characteristics of malaria in Jazan between January 2015 to June 2018 in Aledabi and Baish Jazan Province.
- Explore factors associated with reporting imported malaria cases, and
- Determine the sensitivity of current surveillance classification tool.

## Methods

### Study design

The study used a mixed-method approach: (a) describe the malaria trends using a combination of passive case detection (PCD) and active case detection (ACD) forms used through networks of primary health care centres (PHCCs) and notifications from the private sector to malaria reporting centres, weekly statistical statement, and monthly reporting forms from Baish and Aledabi malaria and vector control from January 2015 to June 2018; and (b) to assess the surveillance system challenges and sensitivity of the current malaria case classification tool used informant interviews (KIIs).

### Study sites

**Fig. 1:**
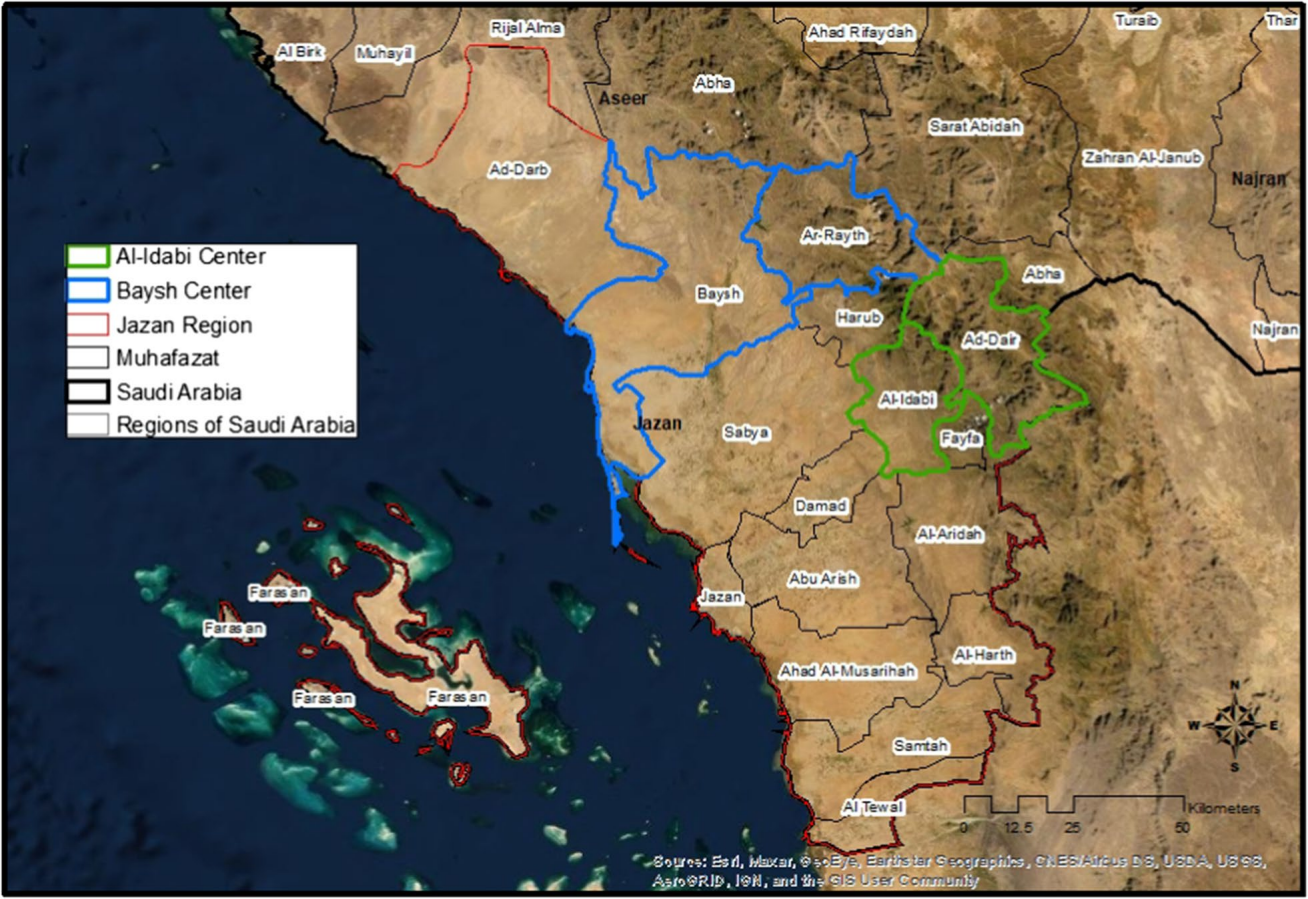
A map of Jazan with the study 1 regions

The two study sites, Aledabi and Baish (Fig. 1), account for more than 70% of all malaria reported cases in Jazan province. Jazan located in the southern part of the Tihamah [25] that stretches 300 km along the southern Red Sea coast, just north of Yemen. Aledabi governorate, also known as the gate of the mountain area, is located at the southern part of the Sarwat mountains (mountain ranges in the western part of the Arabian Peninsula), sharing direct international borders Sa'dah Governorate (Militia Houthi administration area since 2004) in Yemen. In contrast, Baish is in the Baish valley close to the Red Sea coast, the heart of agriculture, and hosting Jazan Economy City (JEC) cities that contain large oil refinery [26].

Together, the two study areas have a total population of around 835, 220 Baish (774,421 people/827 km^2^) and Aledabi (60,799 people/1290 km^2^) out of 1,365,110 total population in Jazan region [27, 28]. The weather in Jazan is subtropical [27, 29]. The average annual maximum temperature is: 32.0 °C. The average annual minimum temperature is: 26.0 °C. The relative humidity is 95% [30]. The annual rainfall in higher land areas of the south experience the highest rainfall in Saudi Arabia, with some parts receiving about 500 mm a year (subject to Indian ocean monsoon occurring usually between October and March) [31].

*Plasmodium falciparum* predominates malaria transmission. The malaria transmission season in Jazan extends from October to April, with a prominent peak in January [32–34]. The primary malaria vector is *Anopheles arabiensis* [35], the only known competent vector for malaria in Tihamah coastal plain in Saudi Arabia] with a sporozoite rate (< 1%) [1], which also peaks following the rainy season [1]. *Anopheles arabiensis* is more abundant in irrigated areas as larvae and as adults than non-irrigated regions of Jazan and showed a high tendency towards anthropophagic behaviour [34]. More than 100 malaria active transmission foci are reported in the Southern region of Saudi Arabia [36].

The local inhabitants are mainly engaged in escarpment agricultural activities, such as growing sorgo, millet, corn, maize, mangoes, bananas, fruits, and vegetables, raising domestic livestock (sheep, goats, camels, and cows), and handicraft work such as ceramics, pottery, and leather goods [25, 34, 37–40]. These agricultural activities attract migrant and seasonal workers from Yemen and East Africa [41]. Traditional houses were constructed of cement block buildings, stone frameworks, and mud structures [25]. In Jazan Province, 63.53% of inhabitants live in traditional housing. Most villages are located along valleys (Wadi) and rarely lie further than one kilometre from a wadi or body of water [25, 30].

Health services, mainly governmental primary health centres, are available and free with laboratory services that can diagnose malaria and provide first-line treatment for locals and transfer complicated cases to regional hospitals [42, 43]. A few private hospitals and clinics exist, but they are obligated to report malaria cases to the malaria elimination programme [37, 44].

### Case definitions and reclassification

Based on Ministry of Health guidelines, imported cases are defined as a malaria case or infection in which the infection was acquired outside the area (international) in which it is diagnosed and was applied in this study analysis [45]. The above working definition was revisited and discussed during the interview with a malaria elimination programme worker to evaluate the current application of classification using travel history as a diagnostic classification tool. To come up with possible reclassification variables that can address border challenges, three key informants were interviewed from each malaria centre. Additionally, one female key informant from the female team selected in each malaria centre was asked to define imported cases and local cases. Informants were also asked about the challenges they might face in classifying the cases including what main obstacles they face, how classification can be improved, and what additional factors might be considered for classification of cases. The following three different definitions of imported malaria were proposed based on the information and feedback from the KII. These included a constructed variable to give three different scenarios of reclassification of imported malaria case:

1. National Malaria Elimination Programme: imported cases were defined as malaria cases or infections in which the infection was acquired outside the area (international) in which it is diagnosed [45].
2. Baish Centre: as malaria case or infection in which the malaria confirmed cases or infections was acquired outside the Saudi international border during a history of travel in the last 10 days for one night or part of the night, while malaria cases contracted locally (i.e., within national boundaries), without travel history from outside of the country (international border) [46, 47].
3. Aledabi (Border area): as malaria case or infection in which the malaria cases or infections in which the confirmed malaria infection was acquired if the patient travelled to a malaria-endemic area in the past month or if there is no evidence of local transmission (vectors or cases live within 500 m radius of the border) [48].

### Data collection and management

Study data were collected originally by the surveillance system, and then entered and managed using Redcap electronic data capture tools hosted at Tulane University. RED Cap (Research Electronic Data Capture). This is a secure, web-based software platform designed to support data capture for research studies, providing (1) an intuitive interface for validated data capture; (2) audit trails for tracking data manipulation and export procedures; (3) automated export procedures for seamless data downloads to common statistical packages; and (4) procedures for data integration and interoperability with external sources.

Extracted variables included date of diagnosis, age, gender, nationality, GPS (of were the case investigation conducted work or living), location of residence and/or work, location of diagnosis, source of information, number of individuals in the patient’s household, whether the area was known foci of active transmission in the last 3 years, travel history (in form of yes or no and details if applicable), date of onset of symptoms, date of diagnosis, diagnostic test used (RDT or microscopy), confirmed microscopy results, patient status (given treatment and stable or given treatment and needs hospital admission), treatment received, case classification as imported or local by health worker, malaria species, confirm the treatment plan and course is completed, previous malaria episode in the last 3 years, positive malaria history in family and neighbours, environmental factors associated with malaria and entomology survey.

### Data analysis

All analyses were done using Stata Statistical Software: Release 16 (2019, College Station, TX: Stata Corp LL). Numerical variables were summarized by means and standard deviations (SDs) if normally distributed and by medians and interquartile ranges (IQRs) if not normally distributed. Frequencies and percentages summarized categorical variables. Pearson’s Chi-squared (χ^2^) test was used to compare proportions, and the two-sample t-test was used to compare means. The Mann–Whitney U-test was used for non-parametric data. All results were considered significant at *p* < 0.05.

Independent variables included in this model include age, gender, living on the border area, non-Saudi nationality, and travel history. The hypothesis posed to the data was that the likelihood that malaria cases in Jazan classified as imported malaria is related to age, gender, nationality, travel history, and being a border resident. A selected logistic regression was performed to calculate the adjusted odds ratio (OR). Five predictor logistic models were fitted to the data to test the research hypothesis regarding relationships between the likelihood that malaria cases in Jazan will be classified as imported according to their age, gender, nationality, travel history, and border residents. A pairwise correlation test was run to detect multicollinearity among independent variables.

The travel history variable was used to conduct a sensitivity analysis on classification. A contingency table was constructed to measure the sensitivity and specificity of the travel history reports against the World Health Organization (WHO) (i.e. the WHO gold standard diagnostic tool for malaria case classification) in the data. The area under the ROC curve also was computed using travel history as a classifier [49]. Finally, a sub-analysis was conducted by the malaria centre to see if there will be any different.

## Results

### General characteristics

A total of 3210 cases with laboratory-confirmed malaria cases were included in the study, 1902 (59.25%) cases were reported from Aledabi centre and 1308 (40.75%) from Baish centre (Table 1). From the 3210 reported malaria cases, 170 (5.30%) were classified as local cases. From the 3040 imported cases, about 90% (2760/3048) cases were males. Yemeni account for 46.00% (1399/3048) of imported cases followed by people from the Horn of Africa (HoA) 17% (505/3048). East Africans migrants are steadily increasing since 2015, 2016, and 2017 from 5%, 16%, and 22%, respectively. Total number of local cases 170 (5.3%), 74% (126/170) were males carries Saudi nationality 94.00% (159/170).

**Table 1:** Basic description of the study population by case classification

	All	Local	Imported
	n = 3210 (%)	n = 162 (%)	n = 3048 (%)
Centres			
Aledabi Centre	1902 (59.25%)	1 (1.0%)	1901 (62%)
Baish Centre	1308 (40.75%)	161 (99%)	1147 (38%)
Age (IQR)	25 (20–32)	24 (17–35)	25 (20–32)
Missing	*3*	*0*	*3*
Sex			
Male	2886 (90%)	126 (74%)	2760 (91%)
Female	324 (10%)	44 (26%)	280 (9.0%)
Nationality			
Saudi	803 (25%)	158 (98%)	645 (21%)
Yemen	1404 (45%)	0 (–)	1404 (46%)
East Africa	507 (16%)	0 (–)	507 (17%)
Other Nat.^a^	122 (4.0%)	4 (2.0%)	118 (4%)
Undefine^b^	374 (10%)	0 (–)	374 (12%)
Travel history			
Yes	2484 (77.38%)	30 (17.6%)	2454 (80.7%)
No	726 (22.62%)	136 (80%)	590 (19%)
Live in border			
Yes	1874 (58%)	6 (3.53%)	1868 (61.47%)
No	1335 (42%)	164 (96.47%)	1171 (38.53%)
Missing	1	0	1
Malaria species			
*P. falciparum*	2734 (85%)	166 (97%)	2568 (84%)
*P. vivax*	464 (15%)	4 (3.0%)	460 (15%)
Other	7 (0.22%)	00 (–)	7 (1.0%)
Mix	5 (0.16%)	00 (–)	5 (0.16%)

^a^Other Nationality includes Indian Subcontinent, Egypt, Sudan, and the Philippines

^b^There are no records of what nationality expect that they are non-Saudi

### Travel history

Seventy seven percent (2484/3210) of the patients spent the night or part of the night outside their village in the last 10 days travelling before the onset of symptoms. Interestingly, 17.6% (34/170) reported travelling outside their place of residence but travelled locally to malaria active transmission foci within the local cases.

### Border area

Around 15. 58% (55/3210) of the reported malaria cases were residents or lived in Saudi–Yemeni borders for more than 10 days. Within the imported points, 16.5% (498/3040) were borderland residents, while 63% (1139/3040) were transient or displaced migrants that are temporary in the border area going to continue further into Saudi Arabia.

### Factors associated with imported malaria

Unadjusted estimates (bivariate regression analysis) were calculated. To obtain adjusted estimates, variables with an unadjusted *p*-value ≤ 0.05 were included in the model. No multicollinearity was detected (i.e., correlation coefficients ≥ 0.50). All variables were fitted in logistic regression to check a possible relationship between factors associated with reporting malaria case as imported malaria (Table 2). Unadjusted estimates were non-Saudi nationality (OR 13.6; 95% CI 8.84–20.8), history of travel (OR 5.76; 95% CI 4.19–7.92) and being border land resident (OR 9.67; 95% CI 7.17–13.04) show a statistically significant association with reporting malaria as imported. Age and sex did not show any association with malaria cases reported as imported (Table 2). Only travel history and border land residence were associated with increased odds ratio of being classified as imported case by malaria programme worker.

**Table 2:** Logistic regressions predicting odds of malaria case reported as imported malaria

	Crude OR	(95% CI)	Adjusted OR	(95% CI)
Age	1.00	(0.98–1)	0.98	(0.97–1)
Sex (male)	3.44*	(2.4–4.9)	1.24	(0.75–2.0)
Nationality (Saudi)	13.5*	(8.8–99.7)	*0.96*	(0.76–1.22)
Border resident	9.6*	(7.2–20.0)	10.5*	(7.42–15)
Positive travel history	5.7*	(4.1–7.9)	1.7*	(1.22–2.4)

*p-value < 0.001

### Sensitivity around classification using travel history as diagnostic tool

Interestingly, 20% (590/3048) of surveillance cases that were reported as imported did not report any travel history to international locations, and the majority were reported by the Aledabi malaria centre. The sensitivity of the travel history as a tool for case classification as imported (malaria cases classified as imported if malaria infection had been traced to international endemic area by reporting history of travel in the last 30 days) in Aledabi reported cases was 68.92% compared to Baish 99.91% (Table 3).

**Table 3:** Sensitivity analysis for travel history as diagnostic tool for malaria classification

	Surveillance		Baish		Aledabi	
	*N* = 3210	95% CI	*N* = 1308	95% CI	*N* = 1902	95% CI
Sensitivity	80.59%	(79–82)	99.91%	(99.5–100)	68.92%	(67–71)
Specificity	80.00%	(73–86)	80.37%	(73–86)	71.43%	(29–96)
Fisher’s exact			0.000		0.034	

The proportions difference between the reclassification of imported cases based on the gold standard (WHO definition malaria case or infection in which the infection was acquired outside the country in which it is diagnosed so we created a variable based on reported travel history regardless to what the surveillance system report) and imported cases as reported in the surveillance records show a statistically significant association (χ^2^ = 337.7; P < 0.001), Baish (χ^2^ = 1.3; P ≤ 0.001), and Aledabi (χ^2^ = 5.28; P = 0.021).

## Discussion

In this study, the data on malaria reported cases from two malaria centres in Jazan region were assessed and different incidence patterns of malaria reporting across this area, and identified risk factors that were associated with imported malaria in Jazan region.

The upsurge of malaria cases in the southern border of Saudi Arabia is not coincidental nor isolated from the political instability in this region. The findings of this study is consistent with the WHO often citing border malaria as a major obstacle for malaria elimination and malaria resurgences in many countries around the world [50–54].

Despite the Kingdom’s success in eliminating malaria from many parts of the country by scaling up vector control activities, increasing access to diagnostic and treatment, the southwest region still struggles from malaria. Jazan has not achieved elimination yet; nevertheless, it has achieved a public health success. The present study reported rising trends of imported malaria cases as well as a worrisome increase in the local malaria transmission and number of active malaria foci.

Identifying the direct causes that contributes to increase in malaria incidence rate is beyond the scope of the current study but permits a more detailed investigation. Nevertheless, multiple genetic population studies in the Arabian Peninsula reported large extent of diversity and parasite genetic structure in Jazan region maybe due to a constant influx of imported malaria infections into the region from surrounding areas in Yemen as infected skilled workers, from endemic areas of Africa and Asia, move to the area in search for a job to secure a better lifestyle [33, 55, 56]. Equally important are the changes in environmental and ecological factors in the last decades. Inclement weather is another factor leading to the increase in malaria cases. Increases in rainfall, precipitation and temperature potentially caused by global climate change has been observed in Jazan leading to a rapid increase in agriculture and economics [31, 57]. Such a phenomenon has resulted in a surge in *Anopheles* vector density, including *An. arabiensis*, especially as observed in irrigation sites [34].

The study reports an increase in reported local malaria cases with time. However, the proportion of imported malaria remains high, which is consistent with previous studies [58]. High proportion of reported malaria infections are among mobile and migrant’s population (MMPs) from malaria endemic area [59–62]. Regardless of the periodic wide mass screening and treatment of asymptomatic individual. The imported asymptomatic parasitemia is often too low to be detected by RDT and microscopy and is only detectable by PCR. Therefore, it can contribute to the ongoing malaria transmission [63], which was observed in other settings [63–68].

Yemeni MMPs account for almost half of the border malaria cases. Another high-risk group that is often overlooked consists of migrants from HoA, and they accounted for approximately 17% of the malaria cases. Reported malaria cases among HoA migrants has been in a steady increase. This observation is consistent with what has been reported related to increased irregular migration form East Africa [69]. There are several reasons that make migrants from HoA which a high-risk group and among the most vulnerable to malaria infection. First, the national malaria elimination programme focused on migrant workers and mobile populations from Yemen, therefore, there is no translated material or targeted intervention to HoA. Second, since the number of HoA residents represent a relatively small part of the migrants’ population and are less likely to seek medical care attention because of their legal status, it is fair to say that this is a very high incidence rate in this group. Third, official statistics do not capture these irregular migration patterns, nor their precise number or the status of migrants after crossing the border; therefore, it is assumed that equivalent, if not more, migrants did not have access to testing and treatment facilities and that they may have carried parasites. Lastly, the study reported that HoA migrants accounted for about 33% of all *Plasmodium vivax* reported malaria infection, a prevalent malaria species in East Africa [70, 71]. Knowing migrants have limited access to health services or malaria control and treatment increases their vulnerability to acquiring *P. vivax* malaria, which might be an important source of the sustained low-level concentration of *P. vivax* malaria in the region. Moreover, the study identified a few locally acquired *P. vivax* cases in active transmission foci: four local cases of *P. vivax* were documented, including a child under 5 years of age with no travel history. Also, local vectors are competent for *P. vivax* in this area, and local *P. vivax* epidemics have been reported previously [3, 39, 72, 73].

According to the results of this study, a significant number of people without clear travel history in the study or history of crossing the border were classified as having imported malaria. This has been documented in the literature as major risk factors for imported malaria were associated with travellers from or through malaria endemic areas in Yemen or lived directly along the border village and often were not citizens [45, 74–77]. The demographics profile in this study was like the patterns reported in other observational studies in border areas in an elimination setting [14, 61, 78–86]. Three are three major themes based on revision of travel history details: frequent travel to known active transmission foci within the Jazan region without using preventive measurements; farming or involvement in animal husbandry; and visits to relatives or employment and frequent commute in border communities.

It was found that travel history has become a less reliable and sensitive tool the closer the case was detected to the border area, nor the current classification is not relevant since border malaria affects local populations even if the infected individuals have not travelled outside such area or across the international border themselves system account for. The sensitivity analysis elucidates that 19.4% of people without clear travel history were classified as imported cases. It is not uncommon in border areas to struggle in classifying cases based on travel history to assess how and where the infection might be acquired [55, 87–89]. Malaria cases in border villages are often classified by national malaria programme workers as imported and described as “imported-border malaria” cases. They were often written as a side note and were unofficially introduced by the malaria programme worker to describe mainly Saudi communities and borderland residents that remain inaccessible to malaria control activities. Many of these border areas are now under heavy military presence for security reasons and coordinates are not possible each time a case presents. On the contrary, detailed travel history within Jazan region helps in identifying active transmission and foci classifications. Further, annual mass blood surveys are undertaken in areas of previous foci. Therefore, very detailed travel histories are required to establish precise locations that were visited during the last 10 days before clinical symptoms. There may be social reasons why respondents might be reluctant to provide accurate information, including recreational travel. These facts make the classification of area-specific incidence hard to define and interpret. However, even with reliable travel histories of those who have been locally mobile, there is no guarantee that an infection can be linked to the travel location or the usual residence.

Despite the remarkable progress toward a malaria-free KSA, there continue to be challenges that have programmatic or pragmatic implications. Currently, data are not always verified immediately, nor are they available soon enough in a digital format for the national programme to verify the information that was provided at the periphery or entry site. Also, reporting forms might not be up to date in all the peripheral centres as result of the constant changes in leadership, which is very common in Jazan. Managers take time to learn, using protocols that often change, how to build experience. Achieving elimination requires retaining involved staff in the various elimination phases in order to sustain new activities, based on accumulated experience, to prevent reintroduction and re-establishment of transmission. So, data driven decisions can be made without affecting the funding and programme staffing. For example, when local malaria cases started dropping earlier in 2014 the fact that more than half of the seasonal workers who used to deliver malaria control activity have been dismissed, resulting in the low number of ITN distribution and fewer vector control activities despite the increased number of active transmission foci.

Another challenge is drug resistance, which often emerges at border areas [90, 91]. Recent surveys in Saudi Arabia and Yemen revealed a high prevalence of drug resistance genotypes *dhfr* and *dhps* mutations associated with SP resistance existed at low prevalence in Jazan area; however, mutations in *Pfcrt* and *Pfmdr1* genes linked with chloroquine, amodiaquine, mefloquine, and possibly artemisinin are common, which is in agreement with a recent report from the area and linked the source of some of them to Africa and Asia [33, 55, 92, 93]. Drug level monitoring is not part of the surveillance nor parasite genotyping. Introducing parasite genotyping helps mapping of onward transmission and provides useful information on drug resistance that guide activities.

Different approaches could be applied to reduce the spread of malaria in places in close proximity to the bordering area. The buffer zone is one such approach. Implementing a buffer zone in and around the bordering area could prevent malaria from entering malaria-free areas.

One health approach is the other important approach that can be implemented to control malaria in the bordering area. The concept of one health in the control of malaria may need collaborative work with various health approaches, including environmental science and human medicine. It can also be established by joint collaboration among governmental, institutional, and nongovernmental organizations. One of the health approaches for controlling malaria is to control the contributing mosquito vector that is involved in the disease transmission cycle. This requires collaboration with the agricultural sector to find an alternative to pyrethroids and enhance the reduction of insecticide resistance. Strong coordination is needed between water, sanitation, hygiene and environmental agencies to control the spread of malaria in communities living in close proximity to vector-prevalent areas. Such an approach would prioritize outbreak prevention by involving local communities in the surveillance of emerging zoonotic diseases, empowering local communities as agents of change rather than relegating them as passive victims [94].

Border areas should be considered a single epidemiological block rather than map drawn borders. Modern-day globalization means that many health issues cannot be resolved by the affected country alone, and this necessitates political consultations, diplomatic negotiations, and cross-border solutions. Building capacity of health diplomacy and borderland resident from Yemen is key for malaria elimination. A buffer area ‘border malaria zone’ (cross-border collaboration used 10 km polio and onchocerciasis programme used 20 km) from both sides of the border [95]. Margins should be chosen based on the local factors that influence malariogenic potential rather than map drawn borders. Such a zone would create the possibility to establish collaboration across countries and overcome the political issue. Cases could be reported to belong to this zone rather than to be pushed to one country’s data, allowing more involvement of indigenous, land border residents and different stakeholders to provide better implantation of control measures with innovative solutions to combat malaria.

Several limitations due to study design and implementation are noted. Firstly, cases are passively detected with a few mass screenings reports with only reports from health facilities not population-control. Secondly, the low sensitivity in classifying cases in surveillance systems operating at the border area because it is hard to be operationalized and tends to resemble high receptivity. Thirdly, in this data set it was not possible to distinguish between case investigation that was initiated as result of case reported to health facilities or case identified during mass screening, as each positive case mandated a case investigation to be initiated that might result in possibly overestimation of R.

## Conclusion

Border malaria is major obstacle for Saudi Arabia in eliminating malaria due to the complex border malaria settings and dynamics. The malaria elimination programme facing many challenges: human movement, migrant populations and cross-border coordination, changing agricultural behaviour, political instability, and national financial commitment. As seen in Jazan, it is fair to posit that human mobility role in increase malaria importation risk. Yet, human mobility is a dynamic system involving multiple demographic groups, localities, and intersecting socio-economic processes that should be addressed as a whole rather than addressing only the migrant mobile population as a risk group posing a threat to malaria elimination. It is essential to evaluate the current diagnostic tools used in the surveillance system because to achieve elimination, more rigorous activity and strategy should take place in the border area to ensure that elimination can be achieved in the long run. It must be recognized that the continuously changing nature of the border area and its high malariogenic potential might threaten the elimination effort if surveillance continues to be suboptimal or weak.

The cordon sanitaire (buffer zone) is a zoning or fencing system that separates the infected and threatened territories. Buffer zones are designed primarily to prevent infected people from moving into clean areas. This can be accomplished by controlling the flow of illegal migrants along the border area. In South Africa, this approach has been widely applied in the fight against several transboundary animal diseases, including African swine fever, foot-and-mouth disease, trypanosomiasis. Implementing a buffer zone around the Jazan region through the Al Mohammed area, where thousands of migrants enter the Kingdom, could be vital to prevent malaria from being imported and infecting the malaria-free area.

Given continuous malaria importation risk, the malaria elimination programme and continued border screening activities are needed, yet they face continuous interruption and limited activities due to the ongoing war at the border. The way forward on how to reduce malaria transmission risk in this complex emergency setting is unclear due to a lack of evidence of the extent to which imported infections and movement dynamics may sustain ongoing local transmission.

There is no available information if the current control measures are sufficiently able to maintain the status of controlled non-endemic malaria. The current operational challenges such as the suboptimal diagnostic tool (travel history) for case classification used in the surveillance system, lack of cross-border coordination, rapid changes in agricultural behaviour, and political instability have not been investigated either [3, 96].

## Data Availability

All the datasets are available on reasonable request to Correspond author.
